# Prospects of Improving Efficiency and Stability of Hybrid Perovskite Solar Cells by Alumina Ultrathin Films

**DOI:** 10.1002/smll.202408435

**Published:** 2024-11-13

**Authors:** Małgorzata Kot, Katarzyna Gawlińska‐Nęcek, Karsten Henkel, Jan Ingo Flege

**Affiliations:** ^1^ Applied Physics and Semiconductor Spectroscopy Brandenburg University of Technology Cottbus‐Senftenberg Konrad‐Zuse‐Straße 1 03046 Cottbus Germany; ^2^ Institute of Metallurgy and Materials Science Polish Academy of Sciences Reymonta 25 St. Krakow 30‐059 Poland

**Keywords:** aluminum oxide, atomic layer deposition, excitons, interface, perovskite solar cells, polarons, stability

## Abstract

Over the last few years, the influence of low temperature (≤80 °C) and, in particular, of room temperature, atomic layer deposited alumina (ALD‐Al_2_O_3_) on the properties of the underlying hybrid perovskites of different compositions and on the efficiency and stability of the corresponding perovskite solar cells (PSCs) is extensively investigated. The main conclusion is that most probably thanks to the presence of intrinsic defect states in the ALD‐Al_2_O_3_ and in the perovskite layers, charge transfer and neutralization are possible and the entire lifetime of the PSCs is thus improved. Moreover, the migration of mobile ions between the layers is blocked by the ALD‐Al_2_O_3_ layer and thus the occurrence of hysteresis in the current density–voltage characteristics of the PSCs is suppressed. Considering the uniform and nondestructive surface coverage, low thermal budget, small amount of material required, and short duration of the established ALD‐Al_2_O_3_ deposition on top of hybrid perovskites, this additional, but fully solar cell technology‐compatible, process step is most likely the most effective, cheapest, and fastest way to improve the efficiency and long‐term stability of PSCs and thus increase their marketability.

## Graphical abstract

A subnanometer‐thick atomic layer deposited alumina at room temperature is shown to be very effective in neutralizing mobile charges and maintaining high performance of perovskite solar cells during their long‐term operation and storage. Thicker alumina films or those deposited at higher temperatures are nearly stoichiometric but are unfavorable for effective charge extraction and transfer due to stronger electrical insulation.

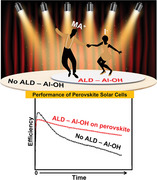

## 1 Introduction

**Figure 1:**
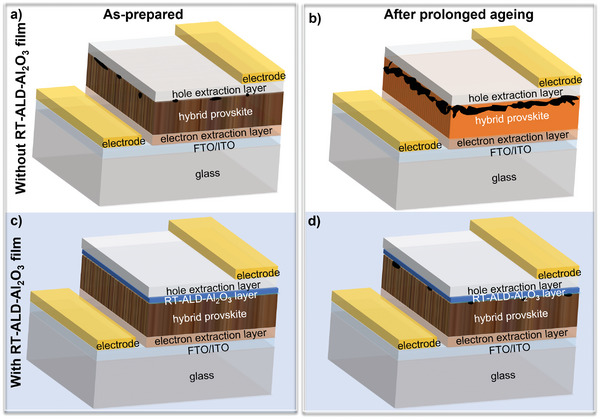
Schematic representation of the as‐prepared a,c) and long‐term aged b,d) perovskite solar cell stack without a,b) and with room temperature atomic layer deposited alumina (RT‐ALD‐Al_2_O_3_) film on top of perovskite absorber.

In recent years, solar energy has drawn an intense attention as the most abundant clean and renewable energy. Many kinds of solar cell devices (e.g., silicon, thin film, organic, organic–inorganic (i.e., hybrid) perovskite) have been developed to convert solar energy directly into electricity.^[^ ^1^ ^]^ Among them, in hybrid perovskite solar cells (PSCs) the perovskite light absorber can be produced in a process of chemical synthesis of very cheap and abundant starting materials, and thus its resources are theoretically unlimited as in the case of silicon. It is also very practical that the PSCs can be prepared in wet‐chemical processes. This means that perovskites can be simply printed onto flexible plastic foils at very low temperatures, reducing thus the total device mass and production costs. That is a great advantage over the commercially available silicon solar cells. The first publication about PSCs dates back to the year 2009, where the authors reported a power conversion efficiency (PCE) of 3.8%.^[^ ^2^ ^]^ After only 15 years of research and development, in the middle of 2024, the certified record PCE of a single‐junction PSC amounted to almost 27%,^[^ ^1^, ^3^ ^]^ that is on a very similar PCE level as the single‐junction silicon solar cells developing since 50′s of the last century. However, despite this rapid PCE development, the stability of PSCs still remains a major obstacle to their market launch. In particular, the perovskite films used in the solar cells architecture (**Figure** **1**a) degrade significantly (Figure 1b, ageing) when exposed to air, moisture, oxygen, ultraviolet (UV) or intense visible light, elevated temperatures, in contact with transporting layers, and under electrical fields.^[^ ^4^, ^5^, ^6^, ^7^, ^8^, ^9^ ^]^ It is well known that defects and surface impurities in active semiconductor materials such as perovskites promote charge carrier recombination, especially at their interfaces with the charge transporting layers, thus also degrading their initial efficiency. As illustrated in Figure 1a, the presence of impurities on the perovskite surface may also result in a discontinuous interface formation with the overlayered charge transporting layer.

Many different methods have been applied to improve the stability and initial efficiency of PSCs. For example, various chemical and mechanical passivation layers and techniques such as organic or polymer layers, two dimensional (2D) materials or laser polishing were used.^[^ ^10^, ^11^, ^12^, ^13^ ^]^ However, very often the solvent‐based post‐preparation treatments do not provide uniform coverage of the perovskite surface thus opening up pathways for further perovskite degradation resulting in reduced long‐term stability.^[^ ^14^, ^15^ ^]^ Moreover, when depositing these passivation layers on the perovskite film, it is also crucial that they can be prepared at relatively low process temperatures (≤80 °C) to avoid damaging the thermally sensitive or unstable perovskite film underneath.^[^ ^16^ ^]^ In addition, the passivation layers must be very conductive or ultrathin in order to allow the transport of the charge carriers generated by the sunlight, i.e., there must still be a sufficient probability that they will tunnel through the passivation layer. The aim of the surface passivation is thus multi‐purpose: (i) to limit the interfacial recombination in particular; (ii) to chemically stabilize the perovskite film surface, i.e., to reduce its reactivity to external conditions; and (iii) to cover the thermally induced micro‐cracks or micro‐holes and thus prevent an undesired charge transport through them (see Figure 1c).

The above mentioned requirements for passivation layers are ideally met by the atomic layer deposition (ALD) technique. In particular, ALD has already been widely used in the production of various types of solar cells.^[^ ^17^, ^18^, ^19^, ^20^ ^]^ It is known, e.g., to provide effective permeation barriers against oxygen^[^ ^21^ ^]^ and moisture.^[^ ^22^, ^23^ ^]^ Besides, ALD precursors can penetrate deep into nanoporous structures, so that high aspect ratio features can conformally be coated by the ALD layer, making it a superior method compared to others.^[^ ^24^, ^25^ ^]^

For the use of ALD layers in PSCs, two main cases have to be distinguished: (I) the ALD process takes place before the perovskite coating, and (II) the ALD coating takes place on the perovskite layer. In case (I), the ALD growth conditions (temperature, choice of metal and oxygen precursors, possible thermal post‐treatment) can be chosen more freely to influence the electronic properties of the charge transport layer (either the ALD layer itself or an underlying film) and to optimize the interface formation with the perovskite material to be deposited on top of the ALD layer. In this process flow, the perovskite layer is not affected by the ALD process that takes place beforehand. The situation changes when the ALD layer is deposited on top of the photoactive perovskite layer (case (II)). Then, the perovskite material and other existing functional layers are subjected to severe thermal and chemical conditions, imposing substantial limitations and requirements with respect to their applicability and stability. For example, halide perovskites readily decompose when exposed to temperatures above 80 °C and/or water molecules.^[^ ^26^, ^27^ ^]^ In this context, the use of water as an oxygen source for the ALD of metal oxides needs to be critically examined. However, it should be noted that the partial pressures typically occurring in the ALD process correspond to a relative humidity of only approx. 0.1%, which is far below the necessary decomposition threshold reported for perovskite layers.^[^ ^28^ ^]^ Therefore, the ALD process using water vapor can be considered promising for the coating of metal oxides on perovskite materials. Rather, more attention needs to be paid to the metal precursor in this regard, as the interface chemistry between the perovskite materials and the metal precursors used in the ALD processes is not yet fully understood.

Conventional wisdom predicts that insulators such as alumina (Al_2_O_3_—please note that, for the sake of simplicity, the formula used here refers to the general name of alumina and not to the stoichiometric form of aluminum (III) oxide) should in principle not be suitable as charge transporting materials due to their large band gaps; however, the band gap and electronic structure of Al_2_O_3_ films deviate strongly from the respective bulk counterpart depending on the film thickness, crystal structure, and the method used for their preparation,^[^ ^29^, ^30^ ^]^ and values between 3.2 and 8.8 eV have been reported.^[^ ^30^, ^31^, ^32^, ^33^, ^34^, ^35^, ^36^, ^37^, ^38^, ^39^ ^]^ Furthermore, as it will be discussed below, ALD‐Al_2_O_3_ films offer a number of advantages and can provide the desired properties to improve the efficiency and stability of PSCs (Figure 1d). To name a few, ultrathin ALD‐Al_2_O_3_ films contain intrinsic electronic defects that are observed throughout the whole electronic band gap^[^ ^29^ ^]^ and are characterized by an extremely low water vapor transmission rate.^[^ ^40^ ^]^ In addition, the vapor pressure of the trimethyl aluminium (TMA) precursor is sufficiently high at room temperature (RT) ensuring a very controlled growth of ultrathin films.^[^ ^41^, ^42^, ^43^, ^44^, ^45^, ^46^ ^]^

According to the Web of Science database,^[^ ^47^ ^]^ the first publication on the application of the ALD technique in PSCs dates back to 2013.^[^ ^48^ ^]^ In that work, Shi et al. investigated organic–inorganic lead triiodide heterojunction solar cells, in which an ultrathin Al_2_O_3_ film was deposited onto methylammonium lead triiodide (CH_3_NH_3_PbI_3_, MAPI) by ALD. However, the authors did not provide any experimental details about the ALD process. Nonetheless, they reported that the ultrathin ALD‐Al_2_O_3_ film significantly enhanced the power conversion efficiency from 3.30 to 5.07%. They speculated that the carrier transport was promoted because the ALD‐Al_2_O_3_ insulating layer (i) contains a high density of built‐in negative charges and thus passivates interfacial defects, (ii) sustains a part of the positive bias applied in the perovskite absorber region close to the back contact, and (iii) decreases the depletion region in the perovskite film close to the back contact. Moreover, they observed that the internal quantum efficiency, especially in the long wavelength range, was improved when between the gold electrode and the perovskite film a thin ALD‐Al_2_O_3_ film was introduced, implying that the free carriers induced in deep levels were collected more efficiently. Last but not least, the authors claimed that introducing an ALD‐Al_2_O_3_ layer between the perovskite and the N^2^,N^2^,N^2′^,N^2′^,N^7^,N^7^,N^7′^,N^7′^‐octakis(4‐methoxyphenyl)−9,9′‐spirobi[9H‐fluoren]−2,2′,7,7′‐tetramin (Spiro‐OMeTAD) hole transporting layer (HTL) in the PSCs, with a mesoporous titanium dioxide (TiO_2_) electron transporting layer (ETL), could block the flow of a shunt current between the ETL and HTL.

The second publication,^[^ ^49^ ^]^ presenting the use of an ALD‐Al_2_O_3_ layer in halide PSCs was published in January 2015. Taking into consideration that the MAPI perovskite tends to degrade in contact with moisture and at elevated temperatures, Dong et al.^[^ ^49^ ^]^ deposited an Al_2_O_3_ film on the MAPI absorber and on the Spiro‐OMeTAD HTL at 70 ^°^C using TMA and ozone (O_3_) as metal and oxygen precursors, respectively. The deposition rate was 0.1 nm per cycle. When the ALD‐Al_2_O_3_ film was deposited directly on the MAPI perovskite absorber, it underwent fast degradation after just one ALD cycle, which had an opposite effect on the performance of the PSCs than expected. On the other side, the use of three ALD‐Al_2_O_3_ cycles on top of the Spiro‐OMeTAD HTL protected the perovskite film from the impact of the aggressive O_3_ oxidant and improved the stability of the PSCs. Nevertheless, the efficiency also dropped significantly in this case, when the number of ALD cycles was increased to five and then to 7. Although a successful improvement in the stability of the PSCs was postulated through the use of an ALD‐Al_2_O_3_ layer, the nature and extent of the effects of passivation, especially in the context of performance degradation, remained unclear.

In the same year, Chang et al.^[^ ^50^ ^]^ used the ALD process of TMA and water (H_2_O) at 100 ^°^C to encapsulate semi‐transparent PSCs. They observed a slight enhancement of the PSCs efficiency (from 10.15 to 10.55%) as well as an improvement of the long‐term stability when a 50 nm thick ALD‐Al_2_O_3_ encapsulation layer was used. However, these authors also did not provide any detailed discussion about the ALD growth mechanism and the properties of the Al_2_O_3_ film.

Few months later, Kim et al.^[^ ^51^ ^]^ proposed a new ALD chemistry for nonhydrolytic ALD‐Al_2_O_3_ processes in which acetic acid and aluminum triisopropoxide as oxygen and aluminium sources, respectively, were used. In all cases, an ALD‐Al_2_O_3_ growth rate of 0.1 nm per cycle at 100 ^°^C was reported. In particular, the authors compared the stability of MAPI films exposed to three different oxygen sources and to three complete ALD‐Al_2_O_3_ processes, all carried out at 100 ^°^C. They used H_2_O and O_3_ based processes with TMA, and the nonhydrolytic one with acetic acid and aluminium triisopropoxide. The authors found that the fastest degradation of MAPI film occurs in the O_3_ based ALD process and the slowest one in the acetic acid based one. However, one should keep in mind that the MAPI exposure to O_3_ was five times longer than to the H_2_O vapor, rendering the conclusion not straightforward. The authors also found that the increase of the Al_2_O_3_ film thickness to 3 and then to 18 nm enhances the lifetime of the MAPI film and slows down its decomposition during annealing at 250 °C. Although an enhanced stability of the MAPI film was presented with an 18 nm thick Al_2_O_3_ film, the authors did not show any influence on the efficiency of the PSCs. In particular, one would expect a beneficial decrease of the energy barrier height and the perovskite depletion width with increasing thickness of the insulating layer; however, one should keep in mind that the Al_2_O_3_ film must not be too thick to warrant the tunnelling of charge carriers through this insulating layer. Therefore, we do not expect any charge flow to the charge transporting layers of the PSC with such a thick Al_2_O_3_ layer.

As can be deduced from the historic development presented above, some attempts were made until the end of 2015 to use ALD‐Al_2_O_3_ in PSCs either as a passivation layer for the perovskite and/or charge transport layers or as an encapsulation layer of the whole device. Important insights notwithstanding, considering the multi‐faceted nature of the “grand” correlation between PSC design, efficiency, and ageing characteristics, this seminal research had to remain incomplete and could not provide a clear understanding of the role of ALD‐Al_2_O_3_ layers in PSCs and especially their influence on the perovskite absorber material.

Motivated by the need to improve the stability of emerging PSCs, since 2015, our group has extensively been investigating the ALD‐Al_2_O_3_/perovskite system by various methods. As mentioned above, a destructive influence of elevated temperature on the stability of the hybrid perovskite films was already reported in the literature since the beginning of the development of PSCs. Therefore, we decided to first study the ALD‐Al_2_O_3_ growth on top of perovskite films at RT. On the one hand, this temperature is much lower than the typical ALD window^[^ ^28^ ^]^ for the ALD‐Al_2_O_3_ process, but on the other hand, a lower negative influence on the properties of the perovskite was expected.^[^ ^41^, ^42^, ^43^, ^46^, ^47^, ^48^, ^49^, ^50^, ^51^, ^52^, ^53^, ^54^, ^55^, ^56^, ^57^, ^58^ ^]^ Specifically, the priorities were set on (i) demonstrating the feasibility of the ALD growth of alumina on perovskite at RT, (ii) investigating the influence of ALD precursors on the morphological, chemical, structural and optoelectronic properties of perovskite layers, (iii) elucidating the ALD growth mechanism on perovskite, and (iv) understanding the impact of the ALD layer on the efficiency and stability of PSCs.

Our first successful application of the thermal ALD process using TMA and H_2_O for the growth of well‐defined Al_2_O_3_ thin films on MAPI at RT has been published in 2016.^[^ ^41^ ^]^ We have found that the TMA precursor and water vapor reactant are chemically active only with the perovskite surface^[^ ^41^, ^54^ ^]^ and that the efficiency of PSCs containing an RT‐ALD‐Al_2_O_3_ layer depends on the Al_2_O_3_ thickness.^[^ ^41^, ^42^, ^46^ ^]^ Moreover, an enhanced stability against ambient air was observed for the perovskite film coated by RT‐ALD‐Al_2_O_3_.^[^ ^41^, ^42^, ^46^, ^54^ ^]^ After that, we successfully demonstrated that the established RT‐ALD‐Al_2_O_3_ process can successfully be used on differently composed hybrid perovskites.^[^ ^41^, ^42^, ^46^, ^54^, ^64^ ^]^ In particular, the RT‐ALD‐Al_2_O_3_ film deposited on an hybrid perovskite is beneficial for a number of processes that can be classified as predominantly structural or electronic interactions.

In terms of structural interactions we found that, the RT‐ALD‐Al_2_O_3_: (i) covers compactly and uniformly the perovskite film,^[^ ^41^, ^43^, ^64^ ^]^ (ii) removes existing defect states,^[^ ^41^, ^57^, ^64^ ^]^ (iii) improves internal stability of the perovskite film,^[^ ^41^, ^46^, ^54^, ^64^ ^]^ (iv) enhances the mechanical adhesion of the overlying charge transport layer with the underlying perovskite, and hence improves the photogenerated carrier collection and transport.^[^ ^42^, ^46^, ^64^ ^]^

Moreover, in terms of electronic interactions, the RT‐ALD‐Al_2_O_3_ film: (v) prevents an additional nanoscale degradation pathway by minimizing the ion migration of the degraded by‐products between the charge transport and perovskite layers in both directions,^[^ ^46^, ^64^ ^]^ (vi) and thus mitigates hysteresis during current density–voltage characterization,^[^ ^46^ ^]^ (vii) stabilizes vacancies in the perovskite film by their interaction with RT‐ALD‐Al_2_O_3_ at the interface.^[^ ^29^, ^43^, ^55^, ^57^, ^64^ ^]^ In particular, polarons and excitons, both intrinsic defects of ALD‐Al_2_O_3_^[^ ^29^ ^]^ and perovskites, play a key role in the interface formation.^[^ ^57^ ^]^

Besides that, (viii) the highest initial efficiency of the PSCs encompassing an RT‐ALD‐Al_2_O_3_ film seems to be related to the completed self‐cleaning process^[^ ^59^ ^]^ of the perovskite surface by the ALD precursors.^[^ ^41^, ^42^, ^46^ ^]^ In particular, we found that the initial power conversion efficiency is even higher than that of the control device (i.e., without an ALD layer) when the RT‐ALD‐Al_2_O_3_ film consists only of tetrahedrally coordinated aluminium ‐ hydoroxy (Al‐OH) bonds^[^ ^43^ ^]^ and no stoichiometric Al_2_O_3_ formation occurs.^[^ ^64^ ^]^ Last but not least, (ix) we have observed that the ALD‐Al_2_O_3_ process enhances the efficiency of the perovskite solar cells over time, even if it gives a lower value initially.^[^ ^41^, ^42^, ^46^ ^]^

Our current work, which has focused on investigating and optimizing other ALD‐Al_2_O_3_ processes on perovskite absorbers at temperatures ≤80 °C, confirmed our earlier assumptions^[^ ^29^ ^]^ that both the use of only TMA pulses during thermal ALD and the plasma enhanced (PE) ALD‐Al_2_O_3_ processes can successfully be used to enhance the performance and stability of PSCs. In the following, we will explain how we have arrived at the above mentioned results and conclusions.

### 1.1 Perovskite Surface Prior to the ALD Process

**Figure 2:**
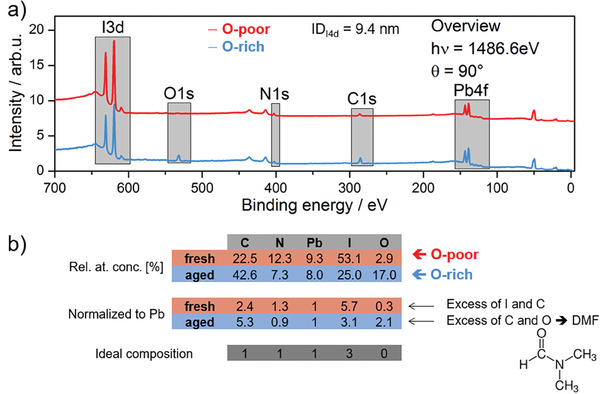
a) X‐ray photoelectron spectroscopy (XPS) survey spectra of methylammonium lead triiodide (MAPI)lead layers investigated with aluminium Kα radiation source (excitation energy of 1486.6 eV) with normal emission geometry (photoelectron take‐off angle, θ = 90°). The information depth (ID) calculated for the I4d core level is 9.4 nm. b) Relative (as calculated and normalized to lead (Pb)) and ideal atomic concentrations of carbon (C), nitrogen (N), lead (Pb), iodine (I), and oxygen (O) calculated from the XPS results. The right‐hand inset shows the structural formula of dimethylformamide (DMF). Adapted from refs. [41, 42].

In the case of ALD, the surface chemistry of the target substrate is of high importance as it determines the growth mechanism of the ALD overlayer. To examine the chemical and electronic properties of the substrate layer prior to the ALD process, we performed X‐ray photoelectron spectroscopy (XPS) on two MAPI films prepared in two different laboratories (**Figure** **2**), which showed slightly different compositions. The first MAPI film was prepared right at the start of the development of MAPI recipes in 2015.^[^ ^41^ ^]^ It contained a significant (actually undesirable) concentration of oxygen (therefore, we call this sample oxygen‐rich, O‐rich), while the second one, produced two years later and in another laboratory,^[^ ^42^ ^]^ was oxygen‐poor (we call it O‐poor hereafter). Considering the binding energy values in the XPS spectra, we suppose that the O‐rich MAPI film must still contain some unreacted dimethylformamide (DMF) used as solvent during the perovskite preparation (see Figure 2b).^[^ ^41^ ^]^ However, as it has been shown for the O‐poor MAPI case, this issue has in the meanwhile been solved during the development of MAPI recipes.^[^ ^42^ ^]^ In addition, both perovskite layers could also have adsorbed some oxygen on their surfaces during their transfer to the ultra‐high vacuum XPS‐ALD system^[^ ^54^ ^]^ in air (see Figure 2). Besides, XPS analysis has shown that the O‐poor film contains an excess of iodine (I) and carbon (C) whereas the O‐rich film is more stoichiometric (only an excess of C and O, that may be related to the mentioned DMF only, was found, see Figure 2b).

### 1.2 Thermal ALD Using TMA and Water on O‐Poor and O‐Rich MAPI Surfaces at RT

**Figure 3:**
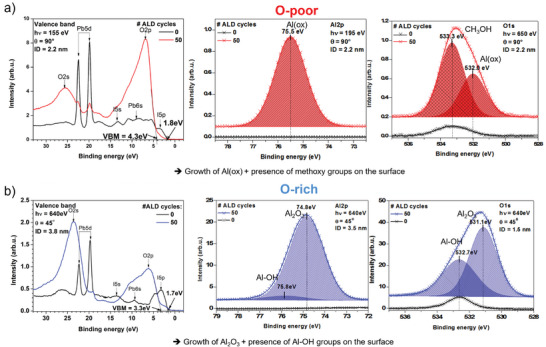
Valence band (VB, left), Al2p (middle), and O1s (right) XPS spectra (measured at the synchrotron) of the oxygen‐poor a) and oxygen‐rich b) MAPI films (black spectra, 0) which were additionally coated with 50 T‐ALD cycles (TMA/H_2_O) at RT (red (a), blue (b) spectra, 50). In (a), the XPS spectra were recorded at a photoelectron take‐off angle of 90° with different excitation energies (155, 190, and 650 eV, respectively), which allowed to probe the same film depth (ID = 2.2 nm). In (b) the spectra were recorded at a photoelectron take‐off angle of 45° using the same excitation energy of 640 eV, resulting in information depths of 3.8, 3.5, and 1.5 nm, respectively. The names of chemical compounds and their binding energies are also indicated. Adapted from refs. [41, 42].

Knowing the surface chemistry of the bare perovskite substrate, we subsequently investigated its influence on the ALD growth mechanism of Al_2_O_3_ on top of it. In the following, the influence of the O‐poor versus the O‐rich MAPI surface on the thermal ALD process of TMA and H_2_O at RT is discussed. The XPS analysis of the ALD growth on both MAPI substrates, using 50 complete ALD cycles consisting of 0.5s TMA, 2s N_2_ purging, 0.5s H_2_O, 2s N_2_ purging pulse times followed by 15s break,^[^ ^41^, ^42^ ^]^ has shown that an aluminium sub‐oxide Al(ox) and methoxy groups (CH_3_OH) are formed on the O‐poor MAPI film (**Figure** **3**a, Al2p and O1s data). In contrast, a nearly stoichiometric Al_2_O_3_ film can be deposited on the O‐rich MAPI surface, containing only a small concentration of Al─OH bonds, which are most probably partly located directly at the interface to the O‐rich MAPI but mainly at the ALD surface (Figure 3b, O1s spectrum). This fact is evident from the much higher signal intensity of OH groups in the more “surface‐sensitive” O1s XPS core level spectra (information depth, ID = 1.5 nm, Figure 3b, right panel) than in the more “bulk‐sensitive” Al2p data (ID = 3.5 nm, Figure 3b, middle).^[^ ^40^, ^42^ ^]^ The presence of the OH groups in the uppermost surface region of the ALD film is very typical for this process. In particular, the ALD process requires free OH groups to enable further growth. We also terminate our ALD cycle with the H_2_O vapor pulse followed by an N_2_ purge pulse. Therefore, the presence of OH groups has its origin also in the ALD process flow. Moreover, it can be seen in Figure 3 (left spectra) that the initial MAPI surface chemistry has a significant influence on the resulting valence band maximum (VBM) position of the ALD alumina film and also on the valence band (VB) spectral line shapes and peak ratios between the signals of the perovskite substrate and the ALD layer, e.g. 5d and O2s or O2p signals. The observed binding energy shifts in the XPS spectra reflect contributions of chemical shifts, electrostatic dipole moments incorporated within the films, and changes in the position of the Fermi energy. The VBM shifts observed in Figure 3 can be attributed mainly to the presence of OH groups in the film and, in addition, to a filling/de‐filling of excitonic states, i.e., charging/recharging processes, resulting in Fermi energy shifts.^[^ ^29^ ^]^ Typically, as soon as the ALD layer growth starts, the XPS signal of the substrate becomes weaker, while that of the growing overlayer increases. From the VB spectral line shapes, we can already conclude that even when using the same thermal ALD process and the same number of ALD cycles, the Al_2_O_3_ film on the O‐rich and O‐poor MAPI substrates grows in different ways or with different kinetics. In the case of the O‐poor MAPI, the substrate signal is more intense, although the XPS measurement was more “surface‐sensitive” (ID = 2.3 nm) than for the O‐rich substrate analyzed with more “volume/bulk sensitive” parameters (ID = 3.6 nm). Assuming the same ALD film properties, the expected result should be reversed. Hence, the deposited alumina films must have different growth rates and subsequently different film thicknesses on the O‐rich and O‐poor MAPI substrates. The corresponding ALD growth mechanism of alumina on O‐poor and O‐rich MAPI films at RT is discussed below.

**Figure 4:**
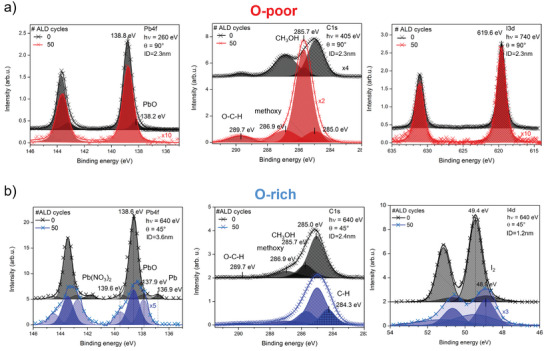
Pb4f (left), C1s (middle), and I3d or I4d (right) XPS spectra (measured at the synchrotron) of the O‐poor a) and O‐rich b) MAPI films, which were also coated with Al_2_O_3_ (50 ALD cycles of TMA and H_2_O) (0: without coating, 50:50 ALD cycles). Please note different scaling factors used in figures that were used to show peaks in the small intensive XPS peaks of the perovskite after ALD. In a), the spectra were recorded with an excitation energy of 260, 405, and 740 eV and a photoelectron detection angle of 90°, which in turn leads to an information depth of ID = 2.3 nm. In (b), the spectra were recorded with a constant excitation energy of 640 eV and a detection angle of 45°, resulting in ID = 3.6, 2.4, and 1.2 nm, respectively. All spectra were deconvoluted and the corresponding binding energies are indicated at each peak. The names of the chemical compounds that are not related to the perfect MAPI perovskite are also indicated. Adapted from refs. [41, 42].

More methoxy and/or OH groups were found in the XPS spectra of the RT‐ALD‐Al_2_O_3_ layer deposited on the O‐poor MAPI film (**Figure** **4**a, C1s data) than on the O‐rich film (Figure 4b, C1s data), suggesting that this ALD process may, in principle, need further optimization for the growth of a pure Al_2_O_3_ film. In the case of the O‐rich MAPI film (Figure 4b), the additional presence of lead oxide (PbO), lead (II) nitrate (Pb(NO_3_)_2_), and iodine gas (I_2_) components was detected in the Pb4f and I4d core level data after the ALD, and these components dominate over the usual MAPI components. Furthermore, a reduction of the metallic lead state was induced by the ALD process. In addition, a new carbon‐hydrogen (C─H) component was found in the C1s spectra for this case.^[^ ^41^, ^42^ ^]^

We would like to note that in our comprehensive XPS characterization of various hybrid perovskites, we sometimes have observed a change of the binding energy and/or the intensity of the peaks during prolonged XPS measurement times. The time needed for changes to occur depends on the used photon flux.^[^ ^58^ ^]^ More photons bring faster changes in the XPS spectra. Therefore, we would like to recommend scientists, who are less experienced in the XPS technique or who outsource XPS studies to other groups, to monitor the XPS spectra during the measurement to avoid misinterpretation of XPS results collected on hybrid perovskites.

Concluding this paragraph, it should be emphasized that only the surface chemistry of the perovskite film was affected by the established RT‐ALD‐Al_2_O_3_ process whereas the bulk properties of both MAPI films were preserved. This statement is further confirmed by X‐ray diffraction results shown for the O‐poor MAPI film in Ref.[42] as well as by other bulk‐sensitive results, to be discussed later in this perspective, for formamidinium methylammonium lead bromide (FAMA) and triple cation (TC) cesium methylammonium formamidinium lead iodine bromide (Cs_0.05_(MA_0.17_FA_0.83_)_0.95_Pb(I_0.83_Br_0.17_)_3_, CsFAMA) perovskites.

### 1.3 RT‐T‐ALD Growth Mechanism of Alumina on O‐Poor and O‐Rich MAPI Perovskite Films

**Figure 5:**
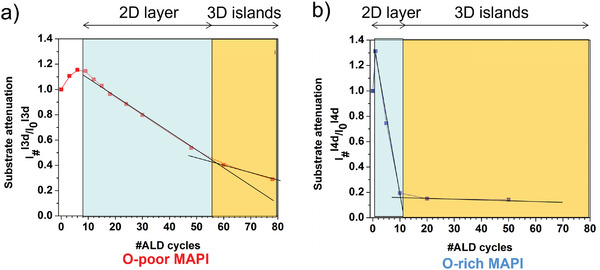
Attenuation of the methylammonium lead triiodide (MAPI) substrate signal (here iodine 3d (I3d) or I4d)) with the number of Al_2_O_3_ atomic layer deposited (ALD) cycles (trimethylalluminium/water, TMA/H_2_O) on oxygen‐poor, O‐poor a) and oxygen‐rich, O‐rich b) MAPI films and determination of the ALD layer growth mode. The information depth in (a) was 2.3 nm and in (b) 3.6 nm. Adapted from refs. [41, 42].

The analysis of the substrate signal attenuation in the XPS spectra can be used to determine the ALD growth mode on MAPI that may follow a layer‐by‐layer, a three dimensional (3D) island, or an intermediate 2D layer followed by 3D island growth.^[^ ^60^, ^61^ ^]^ As illustrated in **Figure** **5**, it does not play a crucial role for the RT‐ALD‐Al_2_O_3_ growth mechanism whether the MAPI substrate layer contains a few or, on the contrary, a lot of OH groups adsorbed on its surface. For both cases, the mechanism seems to be similar, only the reaction dynamics is different. This means that the TMA precursor is very reactive at the beginning of the deposition and has a higher affinity to react with the OH groups and defects present on the MAPI surface than with those of the subsequent H_2_O pulses. In Figure 5, the I3d or I4d signals (substrate signals collected in situ in the ALD‐XPS chamber) increase in the first few ALD cycles. Such behavior has already been observed for other substrates and other ALD processes in the context of in situ XPS‐ALD analysis and is referred to as an initial self‐cleaning process.^[^ ^58^, ^59^ ^]^ Subsequently, after a few ALD cycles, the iodine signal begins to fall off as expected, where for both MAPI substrates, the signal decays very rapidly during a few ALD cycles and much more slowly thereafter, as illustrated by the blue and orange regions in Figure 5. We attribute this behavior to a growth that first occurs in layers and then forms 3D islands. Our analysis shows that the alumina growth on the O‐rich MAPI film (Figure 5b) changes very rapidly from layer to island growth, whereas this occurs much more slowly on the O‐poor MAPI substrate (Figure 5a). Hence, it can be concluded that the initial surface chemistry determines the dynamics of the ALD growth. To verify and confirm the ALD growth mechanism determined from the XPS data analysis, we conducted microscopic studies, i.e., field emission scanning electron microscopy (FESEM)^[^ ^42^ ^]^ and atomic force microscopy (AFM).^[^ ^41^ ^]^ The FESEM results confirmed the 2D layer followed by 3D island growth of T‐ALD‐alumina on the O‐poor MAPI film accompanied by an initial etching/cleaning process.^[^ ^42^ ^]^ The same growth mode was confirmed for the O‐rich MAPI film by AFM, where the root mean square analysis of layer roughness has proven that the smoother layer growth occurs first, followed by the much coarser 3D island growth.^[^ ^41^ ^]^

### 1.4 Intrinsic Defect States in ALD‐Al_2_O_3_ Layers

**Figure 6:**
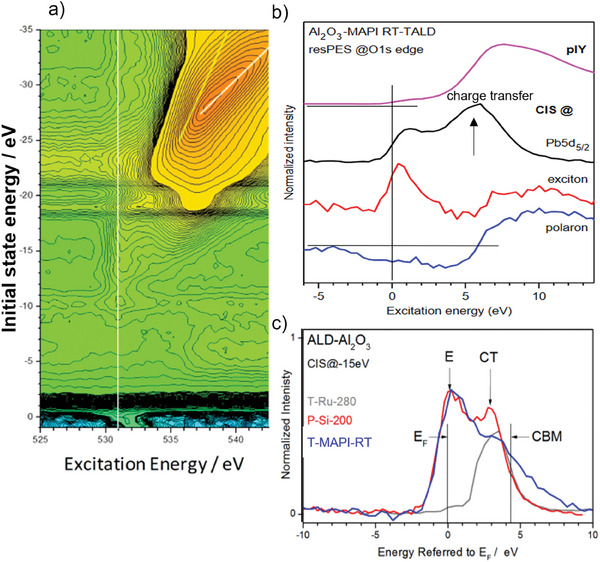
a) Resonant photoemission spectroscopy (resPES) data at the O1s edge of the as grown RT‐ALD‐Al_2_O_3_ film on top of the O‐poor MAPI perovskite. The white vertical lines mark the position of Fermi energy. b) Constant initial state (CIS) cuts, as derived from the resPES data shown in Figure 6a, indicating polaronic, excitonic, and charge transfer states. c) CIS cuts at 15 eV from O 1s resPES data collected on the ALD processed Al_2_O_3_ films on MAPI (blue), silicon, Si (red) and ruthenium, Ru (gray) substrates with marked Fermi energy (E_F_), excitonic (E), charge transfer (CT), and conduction band minimum (CBM). Adapted from ref. [29].

To gain more insights into the electronic properties of the RT‐ALD‐Al_2_O_3_/MAPI interface, we used synchrotron‐based resonant X‐ray photoelectron spectroscopy (resPES, see **Figure** **6**a). A benefit of the resPES technique is its high sensitivity to empty defect states that may exist in the electronic band gap of the material under investigation. In ref. [29], we investigated different ALD‐Al_2_O_3_ processes (thermal and plasma enhanced) performed on different substrates (ruthenium (Ru), silicon (Si) and MAPI) and at different temperatures (from RT up to 280 °C). Regardless of the specific ALD process used, we found that the partial density of states of oxygen 2p (O2p‐pDOS) dominates in the conduction band (CB) for all investigated alumina films. The calculated ratio of the conduction band to the valence band states (N_CB_/N_VB_) is between 8 and 10. This high N_CB_/N_VB_ value indicates that in the Al_2_O_3_ system about 80%–90% of the density of states of the VB is moved into the CB and into the band gap. However, it is generally assumed that only covalent bonding contributions should be considered. We attribute such a high N_CB_/N_VB_ ratio to the existence of localized defect states that contribute to the density of states of both the CB and VB regions. We assume that there must therefore be O2p‐derived defects states that appear within the electronic band gap of ALD‐Al_2_O_3_. In fact, based on the O1s resPES data (exemplarily shown in Figure 6a for the RT‐ALD‐Al_2_O_3_/O‐poor MAPI system) analysis, we found the presence of intrinsic defect states within the electronic band gap of the ALD‐Al_2_O_3_ that we have assigned to polaronic (P), excitonic (E) and charge transfer (CT) defect states (Figure 6b,c). We found that their abundance depends on the ALD process parameters and is different on MAPI, ruthenium, or silicon substrates (Figure 6c). Such defect states are created by interfacial interactions between the substrate and the growing film, with different coordination and bonding geometry at the interface and are localized by Coulomb interactions. Importantly, no external impurities or structural defects are required to create these defect states. We assign the excitonic defect states to the tetrahedrally coordinated and the charge transfer states to the octahedrally coordinated Al ions. In other words, excitonic defect states occur when the aluminum is connected to OH groups, and charge transfer states when it is bound with oxygen. The highest and lowest relative abundance of the excitonic defects in the ALD‐Al_2_O_3_ film is found when it is deposited on MAPI and metallic Ru substrates, respectively (Figure 6c). As both depositions were done by thermal ALD but at different temperatures, we conclude that the number of excitonic defect states is reduced at elevated temperatures. We have shown that the excitonic defect states are related to the density of interface states (D_it_)^[^ ^29^, ^55^ ^]^ which is distinctly decreased after post‐deposition annealing treatment (for example at 450 °C for 30 min).^[^ ^43^, ^62^ ^]^

### 1.5 The Role of Intrinsic Defect States in the ALD‐Al_2_O_3_/Perovskite Interface Formation

**Figure 7:**
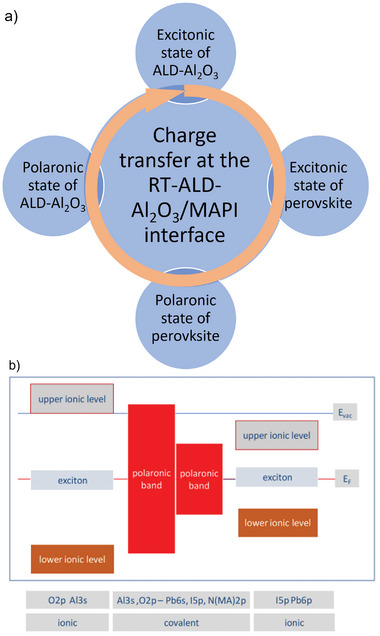
Schematic sketch of the charge redistribution occurring at the interface of RT‐ALD‐Al_2_O_3_ and MAPI a). The orange arrow represents the charge donation from Al into the MAPI and the final back donation into the excitonic state of the stabilizing Al_2_O_3_/MAPI interfacial layer and the respective band alignment. b) Schematic band alignment of the RT‐ALD‐Al_2_O_3_ and MAPI as derived from resPES data. Adapted from ref. [43].

Based on the resPES data analysis,^[^ ^43^, ^57^ ^]^ we developed a model of RT‐ALD‐Al_2_O_3_/MAPI interface formation, in which the intrinsic defect states play a key role (**Figure** **7**). In particular, we found that both interface materials contain intrinsic defect states within their electronic band gaps (Figure 7b). Especially, the excitonic defect states, which are located at the Fermi energy, enable an easy charge transfer between both materials. In our model, the growth of RT‐ALD‐Al_2_O_3_ on MAPI substrate starts from the initial interaction of the TMA precursor with the adsorbed OH groups and/or iodine and methyl ammonium vacancies. We deduce it from the XPS data analysis of the RT‐ALD‐Al_2_O_3_ growth on MAPI substrates, where we always detect an enhancement of the perovskite substrate signal in the first few cycles regardless of the oxidation of the perovskite surface, suggesting its self‐cleaning (see also Figure 5 and related discussion). No pretreatment of the perovskite surface prior the ALD‐Al_2_O_3_ growth is required. As soon as the self‐cleaning process is completed, the TMA starts to react with the oxygen precursor. During the first few ALD cycles the methylammonium (MA) and iodine (I)Po vacancies are attracted toward the MAPI surface and compensate for the charge released by the Al atom (TMA precursor) into the MAPI film. The MA and I vacancies annihilate the electron charges and thereby “fill” the polaronic band of MAPI and then spill over into the polaronic defect band of the RT‐ALD‐Al_2_O_3_ network. From there, these charges can be transferred back to the excitonic defect band of RT‐ALD‐Al_2_O_3_. Both, the Al─O bonding and the MA and I vacancy concentration are stabilized by this complex charge redistribution at the interface. Once the MA and I vacancy pair has reached the corner of the perovskite grain, it can be considered as a nucleus for the formation of holes or cracks between individual grains in the perovskite film,^[^ ^63^ ^]^ which are known to exist in MAPI films. It should also be mentioned that if there are not enough MA and I vacancies in the MAPI film or OH groups adsorbed on its surface before the ALD process, these vacancies may spontaneously be created/attracted on/to the perovskite surface to balance the charge donation from the TMA precursor.^[^ ^63^ ^]^ This fact could explain the differences that we observed in the perovskite morphologies and also in the initial PCE values after the ALD‐Al_2_O_3_ treatment. In particular, as it will be discussed in the following sections, for some perovskites we observed a modified (either reduced^[^ ^42^ ^]^ or improved^[^ ^41^, ^64^ ^]^) efficiency after ALD‐Al_2_O_3_ treatment compared to the control device (w.o. ALD treatment). This is accompanied by the formation of small holes or, on the contrary, an improved image contrast in scanning electron microscopy (SEM) results (improved sample conductivity), respectively. After a certain number of ALD cycles, which corresponds to the end of the self‐cleaning process,^[^ ^42^ ^]^ (see Figure 5), the TMA starts to react only with the H_2_O vapor. The film growth continues with the formation of a continuous layer and then 3D Al–O islands with an increasing number of regularly coordinated octahedral Al sites.^[^ ^43^ ^]^

### 1.6 Influence of the RT‐ALD‐Al_2_O_3_ Layer on the Efficiency and Stability of MAPI‐Based PSCs

**Figure 8:**
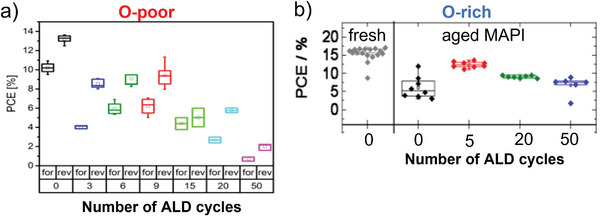
a) PCE in forward (V_start_ < V_end_) and reverse (V_start_ > V_end_) current–voltage scans of the as‐prepared Au/Spiro‐OMeTAD/(RT‐ALD‐Al_2_O_3_)/O‐poor MAPI/PCBM/TiO_2_/ITO/ glass solar cells containing 0, 3, 6, 9, 15, 20 and 50 ALD cycles of RT‐ALD‐Al_2_O_3_ on top of the perovskite film. b) PCE in forward (V_start_<V_end_) current–voltage scans of the as‐prepared Ag/Spiro‐OMeTAD/(RT‐ALD‐Al_2_O_3_)/O‐rich MAPI/TiO_2_/FTO/glass solar cells containing 0, 5, 20, and 50 ALD cycles of RT‐ALD‐Al_2_O_3_ on top of the perovskite film. Adapted from refs. [41, 42].

In **Figure** **8**, the PCEs of Au/Spiro‐OMeTAD/RT‐ALD‐Al_2_O_3_/O‐poor MAPI/PCBM/TiO_2_/ITO/glass (a) and Ag/Spiro‐OMeTAD/RT‐ALD‐Al_2_O_3_/O‐rich MAPI/TiO_2_/FTO/glass PSCs (b) are shown. A varying number of RT‐ALD cycles was applied here. After comparing these PCE trends with the XPS substrate signal decay versus the number of ALD cycles (Figure 5), we conclude that the highest initial efficiency of PSCs containing the RT‐ALD‐Al_2_O_3_ layer is achieved, when the initial substrate cleaning (self‐cleaning) process, induced by the ALD precursors, is completed (left edge of the blue boxes in Figure 5). It corresponds to the point where the I_#_/I_0_ ratio is the highest (I_0_ and I_#_ are the intensities of the signals of the perovskite core level before and after the n^th^‐ALD cycle, respectively, Figure 5). As discussed in the next chapter, our recent investigations on other perovskites corroborate our conclusion that there is a functional dependency between the initial efficiency of the PSCs containing an RT‐ALD‐Al_2_O_3_ layer and the number of ALD cycles employed, i.e., on the thickness of the ALD film. This dependency implies that once the ALD process of alumina on top of a perovskite film is optimized (i.e., preferably only Al‐OH bonds are present), the efficiency of PSCs containing ALD‐alumina layers can be even higher than that of the control device (i.e., without an ALD alumina layer on top of the perovskite film). Therefore, to determine the optimum number of ALD cycles that result in the highest initial efficiency, an in‐situ, cycle‐by‐cycle investigation of the ALD process by XPS to find the highest I_#_/I_0_ ratio is highly recommended.

**Figure 9:**
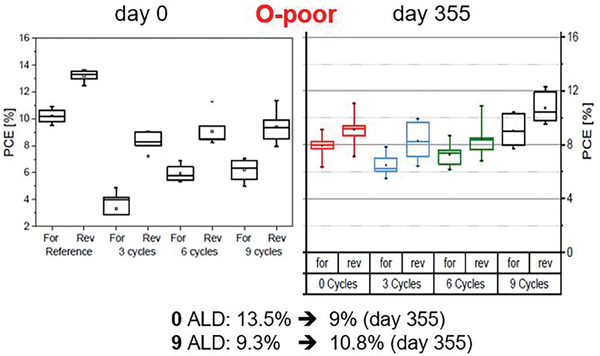
Power conversion efficiency (PCE) of the perovskite solar cells containing O‐poor MAPI film covered by RT‐ALD alumina (TMA/H_2_O) of different thicknesses (applying 0, 3, 6, 9 ALD cycles). Left: directly after the preparation; right: after 355 days of storage in a glove box. Data are shown for forward and reversed directions. Adapted from Ref. [42].

We also find that the efficiency of an aged perovskite film can, to some extent, be recovered by the established ALD process. This conclusion is based on comparing the efficiency of the uncoated fresh (∼15.1%) and aged (∼6.8%) O‐rich MAPI solar cells with the ALD‐coated (e.g., ∼12.4% for 5 ALD cycles) counterparts as shown in Figure 8b. Obviously, the thermal ALD process that we developed using TMA and H_2_O vapor at RT can indeed be used to heal the O‐rich MAPI surface and increase the efficiency of aged PSCs (as also shown for the case of FAMA‐based solar cells below). After the RT‐ALD‐Al_2_O_3_ deposition, the efficiency of the O‐poor MAPI based PSCs is initially lower than that of the bare PSCs, but it increases over time. As shown in **Figure** **9**, after almost one year of PSCs storage (in an argon (Ar)‐filled glovebox with an oxygen gas (O_2_) and H_2_O content of less than 1.0 ppm), the PCE value of the bare MAPI solar cells decreased from 13.5% to 9%, while that of the MAPI/RT‐ALD‐Al_2_O_3_ (9 cycles) increased from 9.3% to 10.8%. Therefore, it can be concluded, that the RT‐ALD‐Al_2_O_3_ film generally contributes to the long‐term healing and protection of the MAPI layers in PSCs.

### 1.7 RT‐ALD‐Al_2_O_3_ Layer on Other Hybrid Perovskites

So far, we have discussed the properties, role, and influence of the RT‐ALD‐Al_2_O_3_ film deposited on the MAPI perovskite. The reason behind this is that MAPI was the most popular and the most studied hybrid (i.e., organic–inorganic) perovskite in the beginning of the PSCs development. However, with the advancement of PSC technology, new and more complex hybrid perovskites have been introduced, ranging from a single (e.g., formamidinium lead triiodide (FAPI)), and going to a double (e.g., FAMA), triple (e.g., CsFAMA) or even to quadruple (e.g., rubidium/cesium/formamidinium/methylammonium (RbCsFAMA)) mixed cation based perovskite solar cells, just to name a few.

We have already studied the influence of the RT‐ALD‐Al_2_O_3_ on MAPI,^[^ ^42^ ^]^ MAFA,^[^ ^46^ ^]^ FAPI [this work], and TCsFAMA^[^ ^64^ ^]^ perovskite materials and corresponding solar cells and found a very similar behavior. In particular, depending on the number of OH groups and defect states available on the perovskite surface, the RT‐ALD‐Al_2_O_3_ treatment may increase (when defect states were removed from the perovskite surface) or decrease (when additional defect states have been created during ALD) the initial efficiency of PSCs. However, in all cases we have observed an improved long‐term stability when employing an ultrathin RT‐ALD‐Al_2_O_3_ film on top of hybrid perovskites.

**Figure 10:**
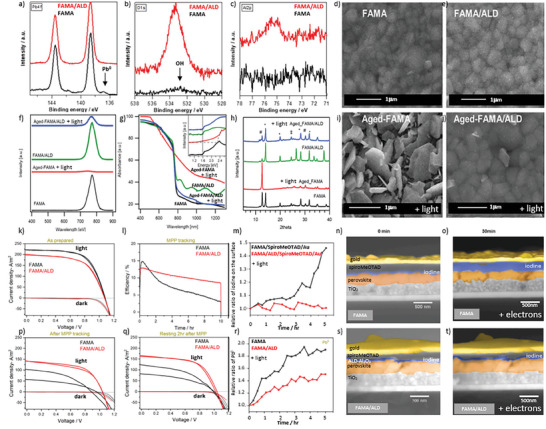
X‐ray photoelectron spectroscopy (XPS) spectra of the Pb4f a), O1s b), and Al2p c) core levels of the 12 months aged (in glove box atmosphere) fromamidinium methylammonium (FAMA) based perovskite film (black spectra) and after deposition of 9 atomic layer deposition (ALD) cycles of trimethylaluminium/water (TMA/H_2_O) at room temperature (red spectra). Scanning electron microscopy (SEM) images of d) 12 months aged and e) covered by 9 cycles RT‐ALD‐Al_2_O_3_, 12 months aged FAMA film. Photoluminescence (PL) f), Ultraviolet‐visible (UV–vis) spectroscopy g), X‐ray difraction (XRD) h) symbols #, *, and ‡ in the diffraction pattern correspond to PbI_2_, perovskite, and formamidinium doped tin oxide (FTO), respectively, and SEM d,e,i,j) results showing differences between reference 12 months aged FAMA film (FAMA) and the same film coated by 9 cycles RT‐ALD‐Al_2_O_3_ (FAMA/ALD) samples after treating them under light in ambient air (Aged‐). Dark and light current density–voltage characteristics k) and efficiency versus maximum power point (MPP) tracking time of the perovskite solar cells with the reference 12 months aged (FAMA) and coated by RT‐ALD‐Al_2_O_3_ (FAMA/ALD) FAMA films l). The current density–voltage (j–V) measurements were repeated after the MPP tracking experiment p) and after resting additional 2h in the dark q). Relative amount of iodine on the gold (Au) surface m) and of the metalic lead defect states (Pb^0^) r) in the FAMA and FAMA/ALD films versus XPS acquisition time. SEM cross section images taken initially n,s) and after 30 min of the SEM characterisation o,t) of the reference n,o) and 9 RT‐ALD‐Al_2_O_3_ coated s,t) 12 months aged FAMA solar cells [Au/Spiro‐OMeTAD/(9‐cycles RT‐ALD‐Al_2_O_3_)/FAMA/TiO_2_/FTO/glass]. Adapted from Ref. [46].

As an example, in **Figure** **10** we demonstrate that the RT‐ALD‐Al_2_O_3_ film can be successfully used to improve the efficiency and long‐term stability of FAMA‐based perovskite solar cells that have already been stored in a glove box atmosphere for 12 month.^[^ ^46^ ^]^ In Figure 10a–c, we show XPS data of the 12 months aged bare FAMA perovskite (black line). We consider the peak located at about 137 eV in the Pb4f_7/2_ XPS core level (Figure 10a, arrow) as a fingerprint of perovskite defects,^[^ ^57^ ^]^ i.e., as Frenkel defect states. After 9 ALD cycles, these defects have been passivated successfully (red line). Furthermore, we found a small concentration of OH groups adsorbed on the FAMA surface (Figure 10b). The peak positions in the O1s (Figure 10b) and Al2p (Figure 10c) data (red lines) indicate that the RT‐ALD‐Al_2_O_3_ film consist of a mixture of Al(ox) and Al─OH. Therefore, we expect a high concentration of excitonic defect states in this RT‐ALD‐Al_2_O_3_ film deposited on the aged FAMA perovskite as well. The morphology of the perovskite film is not significantly affected by the RT‐ALD‐Al_2_O_3_ process, i.e., we did not find holes or cracks in the SEM images after the ALD process (compare Figure 10d,e). Additionally, the used ALD process did not significantly influence either the optoelectronic (Figure 10f,g) or the crystallographic (Figure 10h) properties of this FAMA film (please compare the green and black lines in Figure 10f–h, respectively). Although the initial PCE was slightly reduced after 9 ALD cycles (Figure 10k), the FAMA‐based PSCs with an ultrathin RT‐ALD‐Al_2_O_3_ layer showed improved stability under different stresses. For example, Figure 10i,j shows the SEM images of the FAMA and FAMA/ALD samples after 24 hours of exposure to light in ambient air. Here, the light‐exposed reference perovskite (labeled as “aged‐FAMA”, Figure 10i) is completely destroyed, while the morphology of the ALD protected sample (“aged‐FAMA/ALD”, Figure 10j) sample looks very similar to its untreated counterpart (“FAMA/ALD”, Figure 10e). Besides the FAMA morphology, also the crystallinity (Figure 10h) or optoelectronic (Figure 10f,g) properties are maintained by this ultrathin RT‐ALD‐Al_2_O_3_ layer after prolonged light exposure (compare blue with green results in Figure 10f–h). During tracking of the maximum power point (MPP) (Figure 10l), a slower PCE decay is observed for the FAMA/ALD‐based device than for the bare FAMA‐based PSCs. In addition, the hysteresis in the current density–voltage characteristics typically observed after MPP tracking is significantly reduced when the RT‐ALD‐Al_2_O_3_ layer is added to the PSC stack (Figure 10p). When both samples are left to rest in the dark for 2 h after the MPP tracking, their efficiency increases by approximately the same value, i.e., by about +2%, mainly due to an increase of the short circuit current density (Figure 10q). When characterizing the FAMA perovskite film by XPS, we also found that mobile iodine ions are created by X‐ray irradiation and migrate toward the Spiro‐OMeTAD and Au layers. In contrast, this migration is largely prevented when using the FAMA/ALD layer stack (Figure 10m). At the same time, a higher amount of Frenkel defects (labeled as Pb^0^ in Figure 10r) is found on the FAMA as compared to the FAMA/ALD surface. A similar, but electron‐induced, migration of defects in the FAMA layer, which was also limited by the ALD layer, has been observed during SEM characterization (Figure 10n,o,s,t), where particularly iodine movement is found in the unprotected sample after 30 min of electron exposure (see the wider blue‐colored region in Figure 10o). Based on these results, we conclude that without the ALD layer, the bare FAMA perovskite film gets destroyed, i.e., its morphology and crystallinity are severely disturbed; as also, the efficiency of FAMA‐based PSCs is greatly reduced and a significant hysteresis occurs in their current density–voltage characteristics. However, as soon as the ultrathin (about 0.5 nm thick) RT‐ALD‐Al_2_O_3_ layer is deposited on top of the FAMA film, it contributes to the self‐healing of the partially degraded perovskite by blocking the ion flow through the Spiro‐OMeTAD and Au layers.

**Figure 11:**
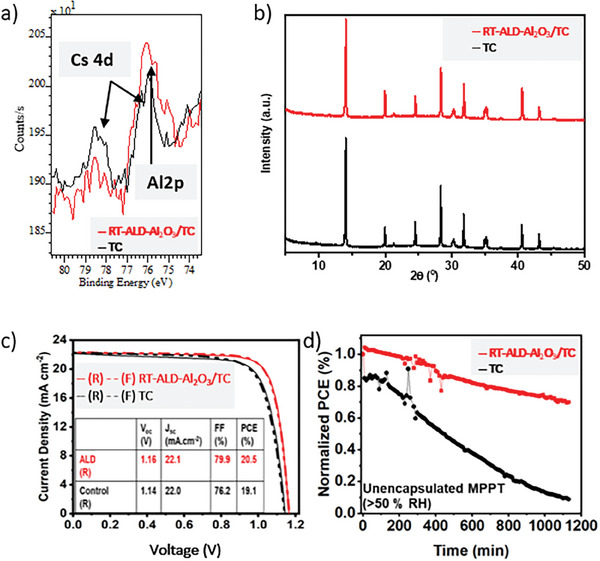
a) X‐ray photoelectron spceroscopy (XPS) results showing the Al2p and Cs4d shallow core levels region, b) X‐ray diffraction (XRD) spectra, c) current–density–voltage characteristics in forward (F) and reverse (R) scan directions and d) a normalized power conversion efficiency versus time of the unencapsulated maximum power point tracking (MPPT) experiment performed on the triple cation (TC) perovskite without (black lines) and with (red lines) 14 RT‐ALD‐Al_2_O_3_ cycles. Adapted from Ref. [64].

As mentioned above, very recently we have investigated the influence of a RT‐ALD‐Al_2_O_3_ film deposited on top of a TC perovskite film on the properties of the related PSCs.^[^ ^64^ ^]^ Unlike in the previous cases, the ALD alumina film was prepared in the industrial ALD system of SENTECH Instruments GmbH^[^ ^65^ ^]^ using modified RT‐ALD process parameters optimized for Si substrates. The Al_2_O_3_ film was deposited on the TC perovskite at RT by using 14 ALD cycles consisting of 60 ms of TMA, 15 s of N_2_ purge, 60 ms of H_2_O, and 15 s of N_2_ purge pulses. According to the calibrated growth rate of alumina on the Si substrate (based on in situ spectroscopic ellipsometry) the resulting thickness should be about 0.75 nm. However, analyzing the Al2p XPS signal of the Al_2_O_3_/TC stack (**Figure** **11**a), we suppose that the ALD film grown on the TC perovskite is much thinner. Our assumption is confirmed by similar findings in Ref. [66], where the authors report about two to four times higher ALD‐Al_2_O_3_ growth rate on Si as compared to a perovskite substrate. It should be noted that in Figure 11a (red line) the Al2p shallow core level of the ALD film overlaps with the Cs4d level of the TC perovskite film. However, it can be seen that the intensity of the Cs4d doublet (peaks located at around 76 eV for Cs4d_5/2_ and 78.5 eV for Cs4d_3/2_ emissions) decreases and the Al2p intensity (peak at around 75.8 eV corresponding to Al─OH, cf. Figure 3b middle) increases after the ALD coating, confirming also the successful growth of alumina. Due to the very low thickness of the ALD film as well as very low concentration (5%) of Cs in the investigated TC perovskite, the signal to noise ratio in this spectral region is also very low. However, the observation that the strongest intensity in this region can be detected after the ALD process at a binding energy of about 75.8 eV suggests that this ALD layer is mostly composed of Al─OH bonds.

Next, in X‐ray diffraction (XRD) patterns of the TC film without and with ALD coating on top (Figure 11b), we again found no negative influence of the ALD process on the structural properties of the perovskite film. Moreover, the current–density–voltage characteristics (Figure 11c), are consistent with our assumption that the highest initial efficiency of PSCs can be achieved after the RT‐ALD‐Al_2_O_3_ treatment when only Al─OH bonds (so preferably no stoichiometric Al_2_O_3_) are present in the ALD film. Namely, the initial efficiency of the champion devices increased from 19.1 to 20.5% after coating the TC film with the thin RT‐ALD‐Al_2_O_3_ (14 cycles). Furthermore, the stability of the TC‐based PSCs has also been significantly improved (see MPPT results shown in Figure 11d) after the RT‐ALD‐Al_2_O_3_ coating. Moreover, we have also found that the RT‐ALD‐Al_2_O_3_ not only blocks migration of ions from the perovskite into the overlayers, but also prevents the migration of the overlayer material (here Spiro‐OMeTAD) into the perovskite film and thus prevents its amorphization.^[^ ^64^ ^]^

### 1.8 Further Developments in the Field of Low Temperature (≤80 °C) ALD‐Al_2_O_3_ on Top of Perovskites for PSCs

To our knowledge, after the first papers mentioned in the introduction, only a few more works (besides the ones including our group) have been published on the application of low‐temperature (≤80 °C) ALD‐Al_2_O_3_ films on different perovskite absorber materials for PSCs applications.^[^ ^28^, ^67^, ^68^ ^]^ Yet, only in one of these papers Al_2_O_3_ growth on a perovskite film was attempted at room temperature. In that particular work,^[^ ^67^ ^]^ the authors investigated the thermal ALD process of alumina on MAPI perovskite at RT, 75 °C, and 125 °C. They also used in situ XPS to monitor the evolution of a representative high‐performance MAPI surface during the ALD process. For the ALD at 125 °C, they observed the formation of metallic lead at the perovskite/ALD‐Al_2_O_3_ interface and a considerable conversion of the bulk perovskite to PbI_2_. As the ALD temperature was lowered to 75 °C and to 25 °C, the degradation was found to be largely minimized, but the authors observed the formation of CH_3_NH_2_. The initial ALD growth at 75 °C was apparently slower than that at 25 °C, indicating that the Al_2_O_3_ film grew more selectively at surface defects, which was desirable as it would not impede charge transport through the interface. Compared to the deposition at 75 °C, the authors, however, found larger deviations in the PSC parameters when the ALD was performed at RT. The ALD at 125 °C was found to most drastically deteriorate the PSCs efficiency. Unfortunately, the impact of the ALD films thicknesses on the efficiency and long‐term stability of PSCs was not discussed by these authors. If the authors had analyzed their XPS results in the same way as we have presented in Figure 5, it would probably have been possible to find the appropriate number of ALD cycles that would ensure the highest efficiency of solar cells for each process temperature. It is possible that using 10 ALD cycles gave the best performance at 75 °C, but it does not rule out that using a different number of ALD cycles at RT would provide an even better result. Therefore, we strongly believe that the use of in situ XPS characterization (or in situ spectroscopic ellipsometry^[^ ^65^ ^]^) during the growth of the ALD films on top of perovskites allows finding the optimum number of cycles (when the initial self‐cleaning process is completed) that, based on our investigations, will give the highest PSC efficiency. Moreover, in our opinion, the formation of metallic lead at the perovskite/ALD‐Al_2_O_3_ interface and a considerable conversion of the bulk perovskite to PbI_2_ observed by these authors could also be related to the exposure of samples to X‐rays, as we reported in Ref. [57]. In addition, analyzing the O1s and Al2p XPS spectra presented in Ref.,^[^ ^67^ ^]^ we observe asymmetric line shapes for all process temperatures, which correspond to the contribution of Al_2_O_3_ and Al‐OH subpeaks. According to our discussion, the later one assures the charge transfer between the ALD and perovskite interface.

Motivated by the observation of strong contributions of excitonic defect states in the resPES data of Al_2_O_3_ films deposited by PE‐ALD process on silicon (see Figure 6c and Ref. [29]), we decided to investigate the effect of PE‐ALD alumina on the efficiency and stability of FAPI‐based PSCs. The appearance of the excitonic defect states indicates that the PE‐ALD‐Al_2_O_3_ film should also be able to compensate for charges on the perovskite surface and that initially the TMA precursor preferably chemically interacts with the perovskite surface rather than with the H_2_O reactant.^[^ ^41^, ^42^ ^]^ Therefore, we prepared sample series in which we performed 10 ALD cycles of TMA, TMA + H_2_O and TMA + O_2_ plasma pulses on FAPI surfaces at 25, 40, 60, and 80 °C (plus 2 cycles of TMA + O_2_ plasma pulses at 60 and 80 °C) and investigated the impact of these ALD layers on the efficiency and stability of the respective PSCs (**Table** **1**). It should be noted that the FAPI surface was coated by an ultrathin 2D octylammonium iodide (OAI) film to stabilize its photoactive α‐phase.^[^ ^69^ ^]^ This work is still in progress but the results are already fascinating and confirm our hypotheses.

**Table 1:** Atomic layer deposited (ALD) film thickness and the power conversion efficiency (PCE) of the octylammonium iodide/formamidinium lead iodide (OAI/FAPI) ‐based perovskite solar cells with and without different ALD treatments and assessed just after solar cell preparation (day 0), after storing them for 45, 90, and 420 days in a nitrogen gas (N_2_) filled glove box. Data are displayed with the highest to the lowest power conversion efficiency (PCE) after 420 days.

OAI/FAPI Treated By	ALD Film Thickness [nm]	PCE at Day 0 (reference) [%]	PCE After 45 Days [%]	PCE After 90 Days [%]	PCE After 420 Days [%]
10 cycles of TMA at 25°C	**0.62**	**13.25**	**18.88**	**19.84**	**18.55**
10 cycles of TMA at 80°C	0.38	13.81	17.58	17.46	17.49
2 cycles of (TMA + O_2_) at 80°C	0.50	17.22	17.00	16.99	16.94
10 cycles of (TMA + H_2_O) at 40°C	0.57	15.11	16.29	18.54	16.83
10 cycles of (TMA + H_2_O) at 80°C	0.38	13.74	17.35	17.62	15.61
10 cycles of (TMA + H_2_O) at 60°C	0.48	11.57	17.35	17.97	15.29
10 cycles of (TMA + H_2_O) at 25°C	0.60	10.74	16.07	12.90	14.63
10 cycles of TMA at 60°C	0.49	15.48	14.12	14.51	14.51
10 cycles of TMA at 40°C	0.59	6.13	13.21	14.77	13.88
no ALD (reference)	**0**	**7.75**	**10.15**	**13.82**	**13.45**
2 cycles of (TMA + O_2_) at 60°C	0.72	14.39	12.47	12.36	11.21
10 cycles of (TMA + O_2_) at 25°C	2.60	6.10	10.10	10.55	9.30
10 cycles of (TMA + O_2_) at 80°C	1.57	6.75	7.44	8.70	9.15
10 cycles of (TMA + O_2_) at 60°C	1.87	3.10	5.21	5.03	8.77
10 cycles of (TMA + O_2_) at 40°C	2.35	2.74	7.81	7.75	7.17

Based on the results summarized in Table 1, several conclusions can be drawn. First of all, the thickness of the alumina grown on OAI/FAPI substrate differs and depends on the used ALD process. In particular, for the same ALD mode (i.e., thermal, plasma enhanced or only TMA) the deposited ALD film is thicker at lower temperature, which is in agreement with stronger material condensation at process temperatures below the ALD window.^[^ ^28^ ^]^

Second, a very similar film thickness prepared by TMA and TMA+H_2_O processes confirms that the TMA initially undergoes a chemical reaction with the surface of the perovskite film rather than with the following water vapor pulse.^[^ ^41^, ^42^ ^]^ This result could open a new research path toward facilitating and accelerating the commercialization of PSCs that most likely do not require an oxidizing step of the TMA precursor to maintain the charge neutralization at the interface, as predicted by us in 2019.^[^ ^57^ ^]^ In particular, we consider the defect‐rich alumina (such as Al─OH or Al(ox)), which contains excitonic and polaronic defect states, sufficient to stop the migration of mobile charged species between the layers it connects within the PSCs stack. Therefore, a modified ALD process using only TMA precursor pulses may be employed to shorten the manufacturing time and protect the perovskite film from contact with harmful water vapor and other aggressive oxidants such as O_2_ plasma.^[^ ^28^ ^]^

Third, the PE‐ALD process grows thicker alumina films on top of perovskite than the thermal ALD process while using the same number of cycles. By reducing the number of PE‐ALD cycles from 10 to 2, the thickness of the PE‐ALD alumina film is successively reduced, which is also reflected in an improved initial efficiency of the PSCs. For example, for the PE‐ALD process performed at 80 °C, the reduction of 10 to 2 cycles reduced the alumina film thickness from 1.57 to 0.50 nm and improved the PCE value from 6.75 to 17.22%, which is the highest initial efficiency (day 0) achieved in this experiment. Moreover, the solar cell containing an alumina layer prepared by 2 PE‐ALD cycles at 80 °C showed the most stable efficiency over storage time. In fact, the PE‐ALD process is known to produce more compact and denser layers than the thermal ALD. We thus suppose that once the PE‐ALD process parameters of alumina are optimized for a specific perovskite, it may eventually lead to even improved long‐term stability of respective PSCs in ambient environment as compared to PSCs containing perovskites overgrown with a thermal ALD layer.

Fourth, the efficiency of the PSCs with the ALD alumina increases over the storage time, which is in agreement with our previous observations.^[^ ^41^, ^42^ ^]^ Interestingly, in contrast to the previous cases, the efficiency of the control device has also increased during the storage period. We suppose that this is due to the presence of the 2D OAI layer on top of the FAPI film that contains NH_3_^+^ and I^−^ ions that may act in a similar way as the RT‐ALD alumina and can also heal the perovskite crystal during storage. We found that the initial efficiency (day 0) continuously increases, and after 90 days it indeed shows the highest value for most of the investigated solar cells. Moreover, for most of the investigated devices this improved efficiency is maintained over another one year of cells storage in the glove box. The best performance after 90 days of storage was found for the PSC with an ALD layer prepared with 10 cycles of TMA only at 25 °C, with a PCE of 19.84%, but the most stable PCE has been observed for the devices containing an PE‐ALD layer prepared by 2 cycles of TMA + O_2_ at 80 °C. Notably, the efficiency of the control device, which has experienced the same environmental conditions (such as storage and transport) as the ALD coated devices, was initially only 7.75%. We would like to emphasize that the goal of these studies was a proof of a concept of the applicability of PE‐ALD to perovskite layers rather than getting a new record efficiency of the PSCs. Once the FAPI‐based PSCs are prepared in the same laboratory without external transportation, the standard PCE value is of about 22%.^[^ ^69a^ ^]^

Regarding the general belief that the PE‐ALD process cannot be used on perovskites,^[^ ^28^ ^]^ we argue that the performance results for the OAI/FAPI based PSCs with the PE‐ALD alumina films shown in Table 1 refute this statement and confirm our strategy that each process should be individually optimized. We would like to mention that the PE‐ALD process of alumina established by us works well also on the MAPI or TC perovskites. In all cases, the key to success was the right choice of PE‐ALD‐Al_2_O_3_ process parameters that neither oxidize the perovskite and nor destroy the C─N bond of the organic molecule.

**Figure 12:**
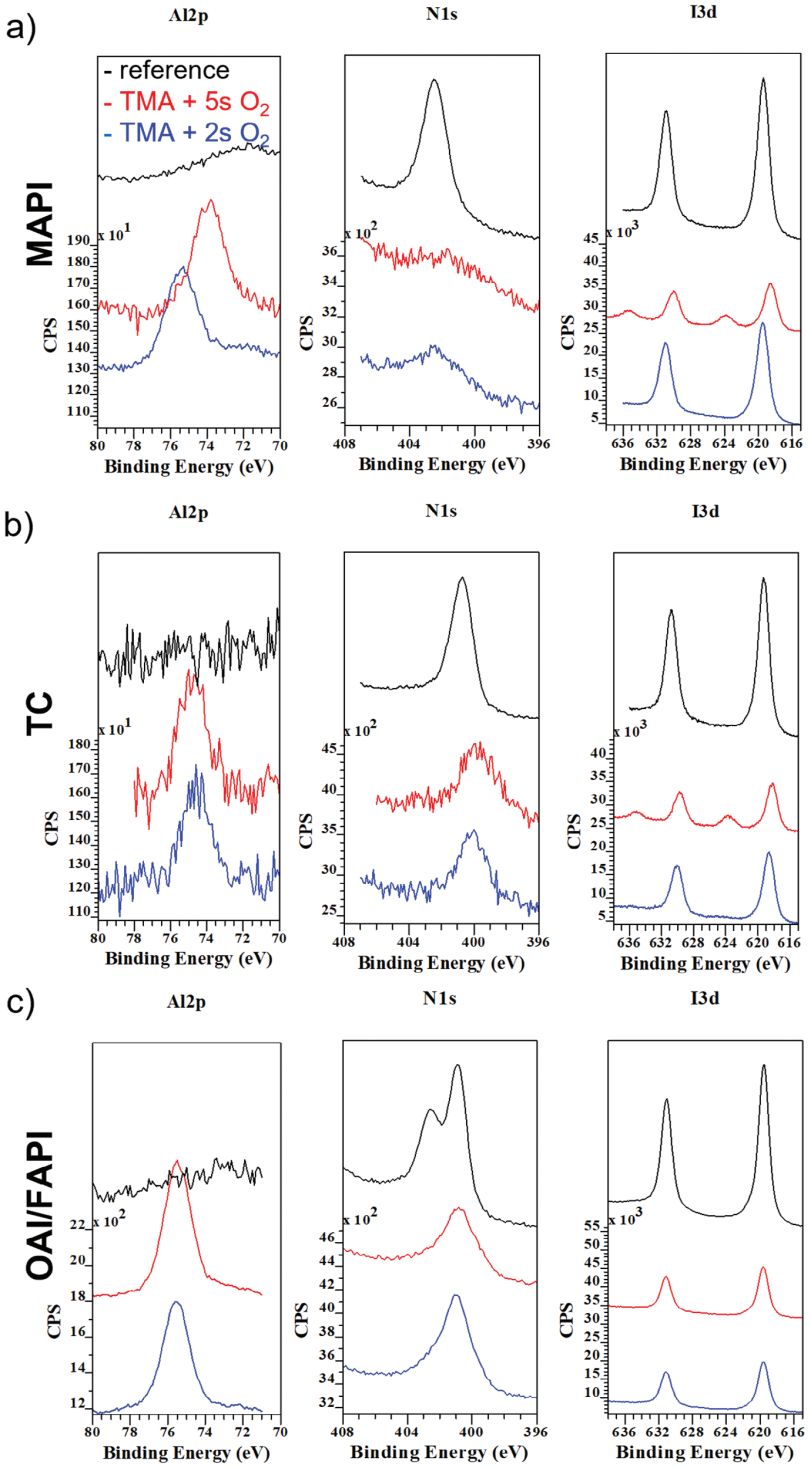
a) XPS results showing the Al 2p (left), N1s (middle), and I3d (right) levels recorded on the reference (black lines) and coated by 5 PE‐ALD cycles of TMA + 5s O_2_ plasma (red lines) or TMA + 2s O_2_ (blue lines) plasma pulses performed on MAPI (a), TC b), and OAI/FAPI c) perovskite films at 60°C. The spectra for each core level were recorded with the same number of sweeps, therefore a lower signal‐to‐noise ratio corresponds to a lower amount of the specific component in that sample. Please note that the spectra for each core level are shown in a waterfall representation.

To prove our statement, in **Figure** **12** we show the XPS spectra of the Al2p (left), N1s (middle) and I3d (right) levels recorded on the reference and PE‐ALD coated MAPI (a), TC (b), and OAI/FAPI (c) perovskites where 5 cycles of TMA and O_2_ plasma pulses of 5s (red lines) and 2s (blue lines) at 60 °C were carried out. As can be seen in Figure 12a, once the PE‐ALD process of alumina is not properly optimized on perovskite (too long O_2_ plasma pulse length, i.e., 5s versus 2s) it can oxidize the iodine at the MAPI surface to I_2_O_5_ (appearance of additional peaks in the I3d level at ∼624 and 636 eV) and destroy the MA bond (weakening of the peak at about 402.6 eV in the N1s region) of the MAPI film.^[^ ^28^ ^]^ Once these changes appear in the XPS spectra, the individual core levels are also shifted toward lower binding energy, in agreement with Ref.[28] Very interestingly, the ALD created I_2_O_5_ peak disappears (data not shown) and the XPS peaks shift back to the expected binding energy values for a given perovskite film within only several minutes of starting the XPS measurement. This phenomenon is possibly based on charging/discharging processes on the perovskite layer, which are triggered by the ALD process and X‐rays resulting in Fermi energy shifts.^[^ ^29^ ^]^

A similar influence of the PE‐ALD process of alumina is found in the XPS spectra of the TC samples (Figure 12b). Apart from the oxidation of iodine by too long O_2_ plasma pulses (see I3d spectra), the FA bond (peak around 400.8 eV in the N1s spectra) seem to be less sensitive to the destructive O_2_ pulses, confirming again the improved stability of this type of perovskite in comparison to MAPI. We did not observe any oxidation of the OAI/FAPI based perovskite film (Figure 12c) even when using a 5s long O_2_ plasma pulse. We found that the peak at around 402.6 eV, which has been associated with the nitrogen bond in the OAI film,^[^ ^69^ ^]^ is also reduced after the PE‐ALD process. Notably, the OAI signal reduction in the XPS spectra has also been observed in Ref.[69]; however, these authors used thermal ALD (TMA/H_2_O) at 100 °C on OAI/FAPI. We suppose that most probably the TMA precursor reacts with the OAI film and reduces it during the alumina ALD process.

Summarizing this section, there are very promising future perspectives for the application of a PE‐ALD process of alumina on various perovskites; however, this ALD process, as well as other ALD processes, must first be meticulously optimized for the targeted perovskite layer.

### 1.9 Other ALD‐Based Metal Oxides, Oxynitrides, and Nitrides Materials for Application in PSCs

As mentioned in the introduction, hybrid perovskites are only thermally stable at lower temperatures (e.g., MAPI is stable ≤80 °C), while the typical ALD window (the temperature range that allows the growth of high quality metal oxides) of thermal ALD processes (using water as oxygen precursor) is between 110 and 300 °C for most metal oxides (see, e.g., Refs.[70, 71]). The ALD window can be shifted to slightly lower temperatures for an oxygen plasma based process (i.e., PE‐ALD), but still it is in a temperature range where perovskite decomposition can be expected. Hence, the application of other ALD‐prepared metal oxides on top of hybrid perovskites is very limited. However, the case is different when the ALD metal oxide is deposited before the perovskite layer. In this case, other metal oxides could possibly be used in the PSC stack.

In the past, we have extensively studied various metal oxides, oxynitrides, or nitrides produced by ALD such as zinc oxide (ZnO),^[^ ^52^ ^]^ aluminum‐doped zinc oxides (AZOs),^[^ ^52^ ^]^ titanium dioxide (TiO_2_),^[^ ^55^, ^56^ ^]^ hafnium dioxide (HfO_2_),^[^ ^55^, ^56^ ^]^ titanium oxynitride (TiO_x_N_y_),^[^ ^72^ ^]^ and indium gallium zinc oxide (IGZO)^[^ ^73^ ^]^ that could potentially also be used in PSCs before the preparation of the perovskite film. For example, in Ref.[52] we studied the influence of ZnO and AZOs ETLs prepared at 200 °C by ALD on top of ITO/glass substrates on the efficiency and stability of MAPI‐based perovskite solar cells. We found that terminating the AZO‐ALD process with the TMA/H_2_O pulse leads to a superior electrical conductivity as compared to the AZO film terminated with a complete cycle comprising a diethyl zinc (DEZ) and a H_2_O pulse or to the pristine ZnO ETLs. Moreover, in the former case, the thermal stability of the sequentially deposited MAPI layer was significantly enhanced and facilitated the charge transport at the AZO/MAPI interface. The corresponding PSCs exhibited a negligible hysteresis and retained 82% of their initial efficiency after ageing for 100 h under ambient conditions with a relative humidity of 40 ± 5%. These results again highlight the importance of atomic layer engineering for the development of efficient and stable PSCs and the crucial role of the TMA/H_2_O‐based ALD process.

Further interface engineering can be achieved by the controlled creation of defects states within the band gaps of oxides, which might contribute to the neutralization of electric charges at the ALD‐oxide/perovskite interface or to a band‐gap narrowing of the oxide increasing the visible light absorption. The analysis of the band schemes derived from the resPES data at the O1s absorption edge for ALD‐prepared TiO_2_ and HfO_2_^[^ ^55^, ^56^ ^]^ revealed that they are very similar to those of Al_2_O_3_ discussed in this work. Hence, the presence of similar intrinsic defect states indicates that these defects must share a common formation mechanism and could contribute to the neutralization of electric charges at the ALD‐oxide/perovskite interface in a similar manner. Furthermore, for TiO_x_N_y_, the band‐gap narrowing was demonstrated by the variation of the ALD process parameters. In particular, we found that a variation of ALD process parameters influences the number of in‐gap states and thus the optical band gap.^[^ ^72^ ^]^

Next, transparent conducting oxides such as IGZO layers are often used as an electron transporting layer in PSCs^[^ ^74^, ^75^, ^76^ ^]^ due to their simultaneous occurrence of high optical transparency and high electrical conductivity, which are actually mutually exclusive. In one of our recent publications,^[^ ^73^ ^]^ we reported on a new PE‐ALD recipe based on a super‐cycle at low temperature (150 °C) for IGZO thin films with an adjustable composition that leads to altered electrical properties. Looking ahead, this low‐temperature PE‐ALD process for IGZO layers could most likely also be performed on a more temperature‐stable perovskite film. Besides that, the termination of the ALD process has a strong influence on the electrical conductivity and the device stability (see above,^[^ ^52^ ^]^). Therefore, exploring the effect of ALD termination of this ternary metal oxide on the performance and stability of PSCs could provide new and fascinating results.

Summarizing the properties of other unary, binary, or ternary metal oxides produced by ALD reported above, we conclude that they can also be used in PSCs, not only as passivating or barrier layers, but also as charge transporting layers. As discussed above, a small change in the ALD process such as process temperature, number of ALD cycles, or pulse length, may indeed translate into a significantly modified device performance. Nevertheless, we emphasize that most of the metal oxides require much higher ALD substrate temperatures than alumina and can therefore most likely only be used in the ALD first/perovskite second sequence of PSC fabrication. For a further study of tentative usability of the ALD method in PSCs and applicable materials, we refer to the literature.^[^ ^44^, ^68^, ^77^, ^78^ ^]^

## 2 Conclusion and Perspectives

One of the key factors limiting mass production of perovskite solar cells is their rather poor long‐term stability. As discussed in this perspective, this obstacle can readily be overcome by depositing a well‐defined alumina passivating layer on top of the perovskite film using atomic layer deposition. Alumina can be grown by ALD even at room temperature with the precursors TMA and water on differently composed hybrid perovskites without any initial surface pre‐treatment. In our understanding, this is envisioned to be based on the charge transfer between defect states found in the resonant X‐ray photoemission spectroscopy data for both perovskite and alumina. The aluminum precursor initially interacts with the adsorbed OH groups and/or with vacancies (methylammonium and iodine) present in the perovskite film. If these vacancies exist in the perovskite film, they will be passivated either by the charge transfer from the aluminum precursor to them or we also assume that the carbon‐based chemical species originating from the TMA precursor will be stabilized at these sites, effectively passivating these vacancies. If there are no free charges on the perovskite surface to compensate for the charge donation from TMA to the perovskite film, these vacancies will be created on this occasion. As a consequence, depending on how clean the perovskite film and its surface are, we can observe an enhancement (passivated defect states) or a decrease (intentional creation of defects to compensate the charge from the aluminum precursor) of the perovskite solar cell efficiencies after the ALD treatment. The same growth trends (initial self‐cleaning followed by 2D layer and 3D islands growth) of alumina were found for different MAPI perovskites, with the only difference being their growth dynamics, i.e., when more defects are present on the surface, a faster transition between the growth phases takes place and less ALD cycles are required. During the ALD growth, the Al bond changes from a more tetrahedrally (excitonic defect state, Al‐OH) to a more octahedrally (charge transfer states, Al─O) coordinated bond. Consequently, the Al─OH or Al(ox) film turns to more bulk‐like Al_2_O_3_, which is actually reflected in the efficiency and long‐term stability of the PSCs, where a thicker ALD film (more ALD cycles) provides longer stability, yet, at the same time, reduces the charge transfer through the transport layers as a thicker film is more insulating. Therefore, an appropriate compromise between the highest possible long‐term stability and acceptable efficiency must be found. The maximal initial solar to power conversion efficiency appears once the initial self‐cleaning process induced by the ALD precursors is completed. Even though the efficiency of the PSCs with the RT‐ALD‐Al_2_O_3_ layer may be initially worse than the efficiency of the solar cells with the bare perovskite, it increases over time, while that of the latter one continuously decreases. Therefore, we recommend reporting the time span between the ALD coating and the PCE measurement in future publications. Based on our experience, after few weeks from the solar cells preparation the PCE of the PSCs with ALD‐alumina film deposited on a perovskite absorber shows the highest values. We believe that the phenomenon of the temporal PCE enhancement of the PSCs with the ALD film can be attributed to intrinsic defect states in the ALD‐Al_2_O_3_ film, which allow continuous compensation of the extra charges generated during device operation and storage.

Our experience clearly shows that thinking outside the box can lead to new and even ground‐breaking results that disprove the basic assumption that coating perovskite surfaces with ALD‐grown insulating metal oxides using water as an oxidant in a RT process should not work. We believe that the fully solar cell technology compatible and “non‐perfect” RT‐ALD‐Al_2_O_3_ process using TMA and water vapor on the perovskite film, which is performed outside the classical ALD window, could already represent a breakthrough paving the way toward commercialization of PSCs. This is due to the fact that an initially slightly poorer performance of PSCs with a passivating RT‐T‐ALD‐Al_2_O_3_ layer compared to PSCs without passivation can be counterbalanced by a significantly better efficiency and stability over time. We would like to note that the RT‐ALD‐Al_2_O_3_ process is not limited to the application in PSCs. Rather, we anticipate that it may successfully be used in many other challenging situations wherever a low‐temperature process must be ascertained, e.g., in applications for flexible electronics, organic materials, and many more, pointing to a broad and promising perspective of applications in these fields. Last but not least, the further optimization of ALD processes and recipes, as indicated in this perspective, can lead to break‐through improvements in PSCs.

## Conflict of Interest

The authors declare no conflict of interest.

## Author Contributions

M.K. contributed in conceptualization, methodology, validation, formal analysis, resources, investigation, data curation, writing—original draft, visualization, and funding acquisition. K.G.‐N. contributed in investigation, data curation, resources, and writing—review and editing. K.H. contributed in writing—review and editing and funding acquisition. J.I.F. contributed in writing—review and editing preparation, and funding acquisition.
